# Quality Improvement Initiative: Utilization of Video Postoperative Instructions in Breast Reduction Enhances Patient Experience

**DOI:** 10.1093/asjof/ojae007.064

**Published:** 2024-04-12

**Authors:** Ruya Zhao, Yi-Hsueh Lu, Daniel Chernovolenko, Aravind Pothula

**Affiliations:** Montefiore Medical Center, Bronx, NY; Montefiore Medical Center, Bronx, NY; Einstein Medical School, Bronx, NY; Montefiore Medical Center, Bronx, NY

## Abstract

**Goals/Purpose:**

The current standard of care for providing postoperative instructions to patients undergoing elective, ambulatory surgeries often falls short in efficiency, clarity, and patient-centeredness. Patients are given a single set of written instructions at the time of discharge after their surgery, which often leads to confusion and increased reliance on phone calls, urgent clinic appointments, and even unnecessary visits to the emergency department. Recognizing this, we propose a quality improvement initiative to enhance postoperative instructions for breast reduction patients through a video format.

**Methods/Technique:**

With institutional IRB approval, all adult patients undergoing bilateral breast reduction (CPT 19318) at an urban academic hospital were included over a 3-month period. A 2-minute video with standardized narrative instructions and visual aids was created. Patients viewed the video during the preoperative visit, and a QR code link to the video was provided in the visit summary on the day of surgery. A Spanish version of the video was available if a Spanish interpreter was used during informed consent. The number of phone calls and emergency department visits within 30 days were recorded and compared to a cohort of patients before this intervention. Qualitative surveys were administered pre- and post-operatively to collect patient feedback.

**Results/Complications:**

During the study period, 59 patients received breast reduction surgery and were given the standardized video instructions pre- and post-operatively (video group). In comparison, 75 breast reduction patients from the prior 3 months, who received nonstandardized, written instructions post-operatively, served as the control group. In the video group, 47% of patients (28 out of 59) made at least one phone call to the clinic, averaging 0.7 calls per patient, while 56% of the control group patients (42 out of 75) made at least one phone call, averaging 1.1 calls per patient. Categorizing reasons for the calls, there was a significantly higher proportion of calls related to wound dehiscence or minor drainage in the video group (41%) compared to the control group (22%; p=0.03). This shift may be attributed to a reduction in routine postoperative care questions due to the clarity provided by the video instructions (FIGURE1). There were 4 unnecessary emergency department visits within 30 days post-surgery in the study period, slightly decreased from the control period's 6 visits (after excluding 1 visit with the patient diagnosed with pulmonary embolism); however, the difference was not statistically significant. Fifteen patients who received the video instruction completed a survey, with 80% rating the video as very helpful, and 80% were able to access the QR code without assistance. These patients scored an average of 81 out of 100 on satisfaction with information measured using the BREAST-Q module after receiving the video instruction. There was no difference in complication between the two groups of patients.

**Conclusion:**

Video instruction proves to be a viable format for patient education and postoperative instruction, offering consistency, multilingual support, and enhanced learning through visual aids. Patient acceptance is high, indicating good baseline digital literacy and encouraging the use of this technology among surgery providers.

